# Texas and Its Measles Epidemics

**DOI:** 10.1371/journal.pmed.1002153

**Published:** 2016-10-25

**Authors:** Peter J. Hotez

**Affiliations:** 1 Sabin Vaccine Institute and Texas Children’s Hospital Center for Vaccine Development (Sabin Vaccine Institute Product Development Partnership), National School of Tropical Medicine, Baylor College of Medicine, Houston, Texas, United States of America; 2 Department of Biology, Baylor University, Waco, Texas, United States of America; 3 James A. Baker III Institute for Public Policy, Rice University, Waco, Texas, United States of America; 4 Scowcroft Institute of International Affairs, The Bush School of Government and Public Service, Texas A&M University, College Station, Texas, United States of America; 5 Scientific Advisory Council, The Immunization Partnership, Houston, Texas, United States of America

## Abstract

Peter Hotez reflects on declining vaccination rates in Texas and the potential for future measles epidemics.

Prior to the introduction of measles vaccine in the early 1960's, serious measles epidemics among school-aged children occurred in the United States every 2–3 years, typically peaking in winter or spring [1]. During that era, an estimated 50,000 hospitalizations occurred annually, together with 500 deaths and 4,000 cases of measles encephalitis, leading to permanent neurologic complications, deafness, or both, as well as billions of dollars in lost productivity and medical costs (Fig 1) [1,2].

**Fig 1:**
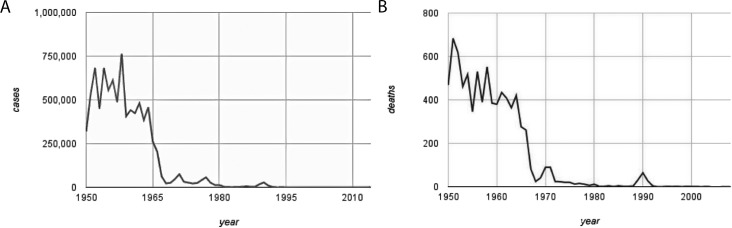
**Decline in US measles cases (Panel A) and deaths (Panel B) since 1950. Original graph, data from US Centers for Disease Control and Prevention:** https://www.cdc.gov/vaccines/pubs/pinkbook/downloads/appendices/e/reported-cases.pdf, accessed September 6 2016.

Globally, the situation was even worse. Measles was one of the leading killers of children, causing millions of deaths annually. Building on the successes of the World Health Organization (WHO)’s smallpox eradication campaign, in 1974, the Expanded Program on Immunization (EPI) was launched, followed by Gavi, The Vaccine Alliance in 2000. Global measles death rates began to decline precipitously. According to the Global Burden of Disease Study 2013, measles deaths decreased 83%, from 544,500 measles deaths in 1990 to 95,600 deaths in 2013 [3]. In the US, measles deaths disappeared [2].

Could large-scale measles outbreaks and deaths return to the US? The measles virus is one of the most highly transmissible human infectious disease agents known, with a basic reproduction number (R_0_) of 12–18 [4]. This number means that a single primary case in a susceptible population would generate on average 12–18 new cases [4]. Because R_0_ is so high for measles, vaccine coverage among a population needs to be extremely high, typically exceeding 90%–95%, in order to prevent a measles outbreak in a school or similar setting [4]. However, the latest numbers from Texas indicate a serious downward trend in vaccine coverage to the point where there is a high risk that measles outbreaks will return.

According to the Texas Department of State Health Services, there are now almost 45,000 children with nonmedical or “reasons of conscience” exemptions to school immunization laws, almost double the number of exemptions in 2010 [5,6] and a 19-fold increase compared to 2003 (Fig 2) [7].

**Fig 2:**
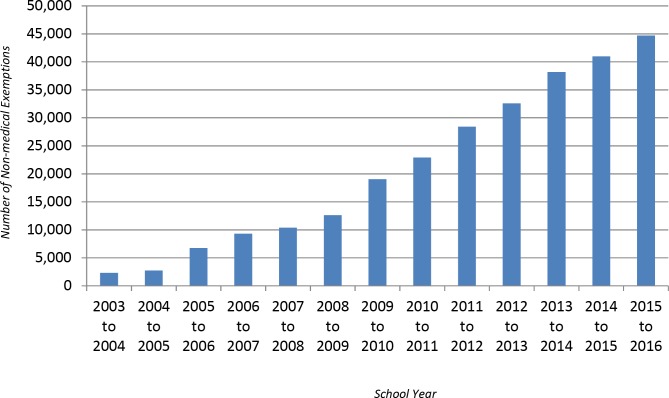
Personal belief exemptions in Texas: K–12th grade students with nonmedical exemptions, Texas, 2003–2016. Original figure courtesy of The Immunization Partnership, 2016. Data from Texas Department of State Health Services, Annual Report of Immunization Status.

Measles vaccination coverage in certain Texas counties is dangerously close to dropping below the 95% coverage rate necessary to ensure herd immunity and prevent measles outbreaks. For instance, in Gaines County in West Texas, the percentage of exemptions is now 4.83%, while in Briscoe County in the Texas Panhandle, the percentage is 3.55% (Table 1) [5]. In the very large Austin Independent School District (Travis County), the exemption rate is at 2.02% [5]. Especially troubling are many of the private schools, mostly in Travis County—the Austin, Texas area—where exemption rates often exceed 20%, including more than 40% of the Austin Waldorf School [6]. The rising numbers of nonmedical immunization exemptions across the state in combination with pockets of very low coverage in vulnerable populations is extremely troubling.

**Table 1:** Rank of leading Texas county vaccine “conscientious exemption” rates (based on information from the Texas Department of State Health Services https://www.dshs.texas.gov/immunize/coverage/Conscientious-Exemptions-Data.shtm).

Rank	County	Area of Texas	Estimated Population, 2012 census^1^	Vaccine Conscientious Exemption Rate 2015–2016
1	Gaines County	West Texas	18,393	4.83%
2	Briscoe County	Panhandle	1,543	3.55%
3	Blanco County	Central Texas	10,673	3.06%
4	Rains County	North Texas	10,962	2.85%
5	Kent County	North Texas	843	2.65%
6	Kendall County	Central Texas	35,968	2.50%
7	Travis County	Central Texas	1,096,246	2.30%
8	Hartley County	Panhandle	6,165	2.24%
9	Burnet County	Central Texas	43,556	2.21%
10	Denton County	North Texas	708,050	2.05%

^1^ http://www.us-places.com/Texas/population-by-County.htm, accessed September 22, 2016.

Although a detailed analysis has not been conducted on the sociology behind the alarming increase in vaccine exemptions in Travis County and elsewhere, a rapidly growing “anti-vaxxer” movement in the state appears to be contributing to the increase in vaccine exemptions. At its epicenter is the Austin-based “Texans for Vaccine Choice,” an organization that describes itself as “a political action committee [PAC] dedicated to protecting vaccine choice rights by ensuring the issue remains at the forefront of political discourse, promoting incumbents and candidates who strongly support our values, and drafting legislation to further solidify these rights” [8]. Their website is set up to take parents step-by-step through the exemption process [9]. Dr. Andrew Wakefield, whose outspoken views and writings alleging links between autism and the measles-mumps-rubella (MMR) vaccine have been refuted by the scientific community [10,11], also now resides in Austin, according to *The New York Times* [12]. Both Texans for Vaccine Choice and Wakefield are heavily promoting the 2016 documentary “Vaxxed: From Cover-Up to Catastrophe,” which was directed by Wakefield and alleges links between vaccination and autism and a cover-up by the US Centers for Disease Control and Prevention (CDC) [13].

In 2015, a study in *The Journal of the American Medical Association* (*JAMA*) of a large sample of privately insured children, comprising more than 95,000 children with older siblings—including 994 (1%) diagnosed with autism spectrum disorder (ASD) and 1,929 (2%) with older siblings with ASD—found “no harmful association between MMR vaccine receipt and ASD even among children already at higher risk for ASD” [14]. Similarly, in that same year, a large case-control study in Japan investigating the relationship between the risk of ASD onset and early exposure to MMR or thimerosal (a mercury-based preservative used in vaccines) also found no link [15], while a 2014 evidence-based meta-analysis of five cohort cases including more than 1.2 million children and five case-control studies including 9,920 children similarly found no relationship between vaccination and autism, nor any relationship between autism and MMR, thimerosal, or mercury [16].

As both a Texas-based research scientist developing vaccines to prevent poverty-related neglected diseases [17] and as a father of an adult child with autism [18], I am also intrigued by data indicating that the neurobiological changes in children with ASD begin early in pregnancy, well before vaccinations are given [19].

Despite the evidence base refuting links between vaccines and autism, as well as a lack of plausibility for such links, the numbers of vaccine exemptions for reasons of conscience continue to increase. We’re at the point at which I believe we might soon see a return of measles outbreaks, possibly far larger than the one that affected a megachurch in Tarrant County, Texas in 2013 [20]. Given that measles peaks in late winter or early spring [1], I predict measles outbreaks in Texas could happen as early as the winter or spring of 2018.

Sadly, the Texas anti-vaxxer movement has become conflated with fringe political elements to create a dangerous and toxic mix of pseudoscience and conspiracy theories. This is now manifesting as a powerful yet misleading, propaganda-filled film documentary, together with an emboldened PAC designed to influence the Texas State Legislature towards anti-vaccine platforms. I worry that, as the most second-most populated state in the US, Texas is seen as a battleground for the anti-vaxxer movement.

But future measles outbreaks in Texas and possible measles deaths are not inevitable. In California, faced with measles outbreaks in Marin and Orange counties, the State Legislature made the bold move of closing loopholes that allow for nonmedical exemptions to vaccines [21]. This measure could prove to be lifesaving in the coming years. We now need to enact something similar for the children of Texas in order to prevent imminent deaths from measles and other vaccine-preventable childhood diseases.

## Abbreviations

ASD: autism spectrum disorder
MMR: measles-mumps-rubella
PAC: political action committee
R_0_: reproduction number
